# Disseminated cryptococcosis in an HIV patient with hepatitis C as the associated risk factor

**DOI:** 10.22034/cmm.2025.345248.1600

**Published:** 2025-03-19

**Authors:** Pallavi Dhawan, Varsha Gupta, Monica Gupta, Parakriti Gupta, Nidhi Singla

**Affiliations:** 1 Department of Microbiology, Government Medical College Hospital, Chandigarh, India; 2 Department of General Medicine, Government Medical College Hospital, Chandigarh, India

**Keywords:** Disseminated cryptococcosis, HIV, HCV, IV drug abuse

## Abstract

**Background and Purpose:**

In the context of HIV/AIDS, cryptococcosis emerges as one of the most common opportunistic infections, with a predilection for affecting individuals with compromised immune function.

**Case Report:**

This study aimed to present a compelling case of disseminated cryptococcosis in a 29-year-old male with a complex medical history, marked by HIV infection, hepatitis C, and a longstanding history of intravenous drug abuse. Blood sample of the patient as well as the cerebrospinal fluid sample grew *Cryptococcus neoformans*. Immunochromatographic test performed on CSF and serum sample was also positive.

**Conclusion:**

Chronic Hepatitis C Virus can disrupt the blood-brain barrier and cause neuroinflammation predisposing the central nervous system to hematogenous seeding during fungemia. Multifaceted medical background of the patient underscored the challenges in the management of comorbidities.

## Introduction

Cryptococcosis is a fungal infection caused by the genus *Cryptococcus* with two species commonly involved in human infections: *Cryptococcus neoformans* and *Cryptococcus gattii*. It poses a formidable challenge in infectious diseases, particularly among immunocompromised individuals. Spectrum of cryptococcal disease ranges from localized pulmonary involvement to disseminated infection, with the latter presenting a significant clinical concern due to its potential for severe morbidity and mortality [ 1 ]. Disseminated cryptococcosis occurs when the pathogen spreads beyond the lungs to involve various organs, including the central nervous system (CNS), leading to meningitis, making early recognition and intervention paramount in patient management. The interplay between Human Immunodeficiency Virus (HIV)-induced immunosuppression and the pathogenicity of *Cryptococcus* species underscores the importance of vigilance in the recognition and management of this potentially life-threatening condition [ 2 , 3 ].

Through a comprehensive examination of this case, this study aimed to elucidate the clinical features, diagnostic modalities, and therapeutic strategies employed in the management of disseminated cryptococcosis in a patient with co-infection of HIV and Hepatitis C Virus (HCV). This case highlighted the role of prompt diagnosis and tailored antifungal therapy in the achievement of favorable clinical outcomes. Additionally, it serves as a reminder to consider opportunistic infections in the differential diagnosis of immunocompromised patients and emphasizes the necessity of a multidisciplinary approach to patient care, integrating infectious disease expertise with specialized knowledge in HIV/Acquired Immunodeficiency Syndrome (AIDS) management and substance abuse rehabilitation.

## Case Report

A 29-year-old male presented to the emergency department with complaints of abdominal pain predominantly in the right hypochondrium associated with loose stools (3-4 episodes/day). Additionally, he had a high-grade fever that persisted for two weeks. The patient had a history of intravenous drug abuse for three years and was known to have coexisting HIV and HCV infection. There were no reported symptoms of chest pain, cough, hemoptysis, nausea, vomiting, diarrhea, or neck swelling. He had experienced one episode of melena 15 days prior but did not seek medical attention.

Upon examination, the patient exhibited fever (100 °F) and severe pallor and was also malnourished with a body mass index of 19 kg/m^2^. There was evidence of terminal neck rigidity but no other significant abnormalities were observed on neurological examination. The patient remained lucid and responsive. He did not have oral thrush, oral/genital ulcers, or any significant lymphadenopathy. Fundus examination showed grade 2 papilledema and abdominal examination revealed mild hepatosplenomegaly. 

The chest radiograph was non-contributory. Abdominal ultrasound revealed hepatosplenomegaly with a heterogenous pancreas. Although he had a history of infective endocarditis, the transthoracic echocardiography was normal. Weekly trends of hematological and biochemical parameters are shown in Table 1. Haemoglobin ranged from 5.9 to 7.2 g/dL initially, and there was thrombocytopenia for which he received four units of random donor platelets. Renal function tests were deranged with urea and creatinine peaking at 149 mg/dL and 4.0 mg/dL respectively. Elevated liver enzymes [alkaline phosphatase (ALP), aspartate aminotransferase (AST), and alanine aminotransferase (ALT)] were noted on more than two occasions attributed to HCV infection and acute liver injury due to systemic infection. There was no evidence of intrahepatic or extrahepatic obstruction. Low protein and albumin levels were consistent with the underlying conditions.

**Table 1:** Weekly trend of the haematological and biochemical parameters

Name of parameter	On date 14.3.2024	On date 23.3.2024	On date 1.4.2024 (after 2 weeks of anti-fungal therapy)	Reference value
Haemoglobin	7.2	6.2	8.8	12-18 g/dl
PCV	23	20	27	36-54%
MCV	84	82	89	80-96 fL
Platelets count	17	43	65	150k-450 k/µL
Reticulocyte%	-	2.6	4.55	0.2-2%
TLC	12.04	8.2	6.58	4k-11 k/µL
DLC (N/L/E)	59.3/15/11.5	79.4/16.6/4	71.8/17.8/8.1	N- 40-75%, L- 20-45%
Sodium	137	142	137	135-145 mEq/L
Potassium	4.3	4.2	4.6	3.5-5.5 mEq/L
Chloride	107	111	103	98-107 mEq/L
Urea	99	136	108	15-45 mg/dL
Creatinine	3.7	2.4	2	0.80-1.80 mg/dL
Bilirubin (Total/Conjugated)	0.8/-	-	0.4/-	Total-0.2-1.0 mg/dL, Conjugated-0-0.25mg/dL
ALP	748	618	532	40-130 IU/L
AST	103	25	57	5-40 IU/L
ALT	42	20	42	5-40 IU/L
Total Protein	7.6	6.7	5.2	6-8gm %
Albumin	2.1	1.7	2.4	3.8-5.5 gm/dL
PT	15	-	-	12-15 seconds
aPTT	50	-	-	28-32 seconds
Amylase/lipase	62/43	-	-	22-80 IU/L/13-60 IU/L

PCV: packed cell volume, MCV: mean corpuscular volume, TLC: total leukocyte count, DLC: differential leukocyte count, ALP: alkaline phosphatase, AST: aspartate aminotransferase, ALT: alanine aminotransferase, PT: prothrombin time, aPTT: activated partial thromboplastin time

**Figure 1:**
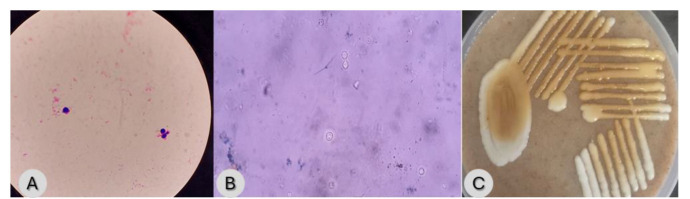
A: Gram-stained smear showing Gram-positive budding yeast cell with pink halo suggestive of a capsule. Figure 1B: India ink preparation showing budding yeast cells with capsule suggestive of *Cryptococcus* sp. Figure 1C: Brown-colored colonies on Bird Seed Agar.

Blood culture samples were sent for routine culture and sensitivity. An automated blood culture system (Biomérieux BACT/ALERT® 3D) detected growth after overnight incubation. Direct Gram stain from the positive flagged bottle revealed gram-positive yeast cells (7-8 µm) with capsulated appearance. India ink preparation was also made which was positive for capsulated budding yeast cells suggestive of *Cryptococcus* sp. (Figure 1). Subculture on Sabouraud’s dextrose medium grew creamy pasty mucoid colonies (incubated aerobically at 37 ℃ overnight) which were confirmed to be *Cryptococcus* sp. on bird seed agar (HiMedia). On bird seed agar, *Cryptococcus* sp. produces brown pigmented colonies due to melanin under the action of phenol oxidase enzyme (Figure 1).

Lumbar puncture was initially withheld due to thrombocytopenia but was performed after improvement. The cerebrospinal fluid (CSF) analysis confirmed *Cryptococcus* sp. and matrix-assisted laser desorption ionization time-of-flight mass spectrometry (MALDI-TOF MS, VITEK® MS PRIME, Biomerieux) identified the isolate as *C. neoformans*. Cryptococcal antigen lateral flow assay (CryptoPS, Biosynex, Strasbourg, France) on serum and CSF was also positive, supporting the diagnosis. 

### Treatment and outcome

The patient was administered liposomal amphotericin B (5 mg/kg per day) and fluconazole (800 mg daily) for 14 days. Renal and hepatic functions along with electrolytes were closely monitored. Renal function gradually improved and hepatic enzymes, particularly ALP, showed moderate improvement. The patient responded well during hospitalization with improvement of neurological symptoms. At discharge, he was advised to continue fluconazole (400 mg daily for another eight weeks) and follow up in the antiretroviral therapy (ART) clinic. He was started on highly active antiretroviral therapy (HAART) and HCV treatment, post-anti-fungal therapy and demonstrated clinical improvement.

## Discussion

Cryptococcosis is a significant global health concern, particularly among HIV/AIDS patients, accounting for approximately 152,000 deaths in 2020 due to cryptococcal meningitis [ 4 ]. The burden is higher in low-resource regions, like sub-Saharan Africa and parts of Asia, including India, where gaps in ART coverage, delayed diagnosis, and limited access to antifungal medications contribute to higher mortality rates [ 4 , 5 ]. India reports an estimated 11,526 annual cases of cryptococcal meningitis, predominantly linked to advanced HIV disease [ 5 ]. High-resource settings, with robust ART programs and early screening, have lower prevalence rates, underscoring the need for global health equity to combat this preventable disease [ 4 , 5 ]. 

*Cryptococcus neoformans* employs strategies to evade the immune system, including a polysaccharide capsule and melanin production. The capsule inhibits phagocytosis, reduces antigen presentation, and suppresses inflammatory cytokines, blunting the immune response. Melanin neutralizes oxidative stress, diminishes cytokine reactivity, and reduces antifungal treatment efficacy, enhancing virulence [ 6 ]. 

This immune compromise is compounded in individuals with HIV/AIDS, where *C. neoformans* often thrives. The HIV-induced immunosuppression significantly increases the risk of opportunistic infections, with cryptococcosis being considered an AIDS-defining illness. The presented case aligns with existing studies demonstrating the heightened susceptibility to disseminated cryptococcosis in HIV-infected individuals, highlighting the importance of early recognition and treatment in this population [ 7 , 8 ].

Diagnosis of cryptococcosis in patients with HIV, HCV, and IV drug use is challenging due to overlapping clinical features with other opportunistic infections, such as tubercular or bacterial meningitis. Cryptococcal antigen testing, particularly lateral flow assay, offers good sensitivity and specificity facilitating early detection in serum and CSF. These tools provide evidence especially in resource-limited settings [ 9 ]. 

Coexistence of HCV infection and IV drug abuse complicates cryptococcosis management in HIV patients. Immunomodulatory effects of substance abuse coupled with HCV-induced hepatotoxicity, compromise immune function. Liver disease reduces T cell response, lymphocytic activity, and complement components, like C5b-9, lowering antimicrobial defense [ 10 ]. Chronic HCV can disrupt the blood-brain barrier and cause neuroinflammation predisposing the CNS to hematogenous seeding during fungemia [ 11 ]. El Serag et al. [ 12 ] found that patients with HCV infection had a significantly higher prevalence rate of Cryptococcal infection, compared to the controls (0.4% vs. 0.1%). Decompensated liver disease is also an important risk factor for the dissemination of cryptococcal infection. Baddley et al. [ 13 ] reported that cirrhosis (due to any cause, including HBV or HCV infection) leads to a 5.8-fold increase in the risk of extrapulmonary dissemination of cryptococcal infection. Liver disease patients develop collateral circulation which allows *Cryptococcus* sp. to bypass the liver scavenger system and lead to spill over into circulation and hence, dissemination [ 14 , 15 ]. The IV drug use alters immune responses, further heightening susceptibility to infections [ 10 , 16 , 17 ]. The HCV infection in IV drug users is present in 60-80% of cases [ 18 ]. The HIV and HCV co-infection is common, sharing transmission routes, like IV drug use.

Prompt initiation of liposomal amphotericin B therapy aligns with current recommendations for disseminated cryptococcosis. Three-phase treatment approach of WHO, consisting of induction therapy with liposomal amphotericin B, flucytosine, and fluconazole for rapid fungal clearance, followed by consolidation and maintenance with fluconazole is effective but challenging in low-resource settings due to financial and logistic barriers [ 19 , 20 ]. Patients with HIV and liver disease are particularly vulnerable, facing heightened risks of nephrotoxicity from amphotericin and hepatotoxicity from fluconazole. Similarly, IV drug users encounter unique adherence challenges and complications due to co-occurring infections and substance use disorders. Tailored treatment strategies and integrated care models are essential for overcoming these challenges, as demonstrated in this case.

## Conclusion

This case emphasized the need for a multidisciplinary approach in the management of patients with complex medical histories, particularly those with IV drug use and multiple comorbidities. Early recognition and treatment of opportunistic infections, such as cryptococcosis, are essential in the improvement of patient outcomes and reduction of morbidity and mortality associated with these conditions.

## References

[1] Maziarz EK, Perfect JR ( 2016). Cryptococcosis. Infect Dis Clin North Am.

[2] Madhavan A, Sachu A, Samuel A, Vasudevapanicker J ( 2022). Cryptococcal antigen prevalence in HIV patients from a tertiary care centre in South India. Iran J Microbiol.

[3] Okurut S, Boulware DR, Olobo J, Meya DB ( 2020). Landmark clinical observations and immunopathogenesis pathways linked to HIV and Cryptococcus fatal central nervous system co‐infection. Mycoses.

[4] Kabir Z, Cunningham C ( 2022 ). The global burden of cryptococcosis—a neglected tropical disease?. Lancet Infect Dis.

[5] Ray A, Aayilliath K A, Banerjee S, Chakrabarti A, Denning DW ( 2022). Burden of sSerious fungal infections in India. Open Forum Infect Dis.

[6] Yang C, Huang Y, Zhou Y, Zang X, Deng H, Liu Y, et al ( 2022). Cryptococcus escapes host immunity: What do we know?. Front Cell Infect Microbiol.

[7] Bramantono B, Danial A, Hadi U ( 2020). A case of an AIDS patient with Cryptococcus neoformans infection. Pan Afr Med J.

[8] Longhitano A, Woolley I, Upjohn L, Korman T ( 2022). New diagnosis of HIV with Cryptococcus neoformans infection presenting as a pleural syndrome. AIDS.

[9] Dantas KC, de Freitas—Xavier RS, Spina Lombardi SCF, Júnior AM, da Silva MV, Criado PR, et al ( 2023). Comparative analysis of diagnostic methods for the detection of Cryptococcus neoformans meningitis. PLoS Negl Trop Dis.

[10] Lozada-Ramos H, Álvarez-Payares J, Daza-Arana JE, Salas-Marín LM ( 2024). Cryptococcal Meningitis in an HCV-positive and IVDU- and HIV-negative patient: A case report and literature review. Int Med Case Rep J.

[11] AI Akhrass F, Madison Tackett S, Mikhael E, Akhter S, Hanif SNM ( 2020). Central nervous cryptococcosis and chronic hepatitis C: two case reports and review of the literature. Clin Surg.

[12] El-Serag HB, Anand B, Richardson P, Rabeneck L ( 2003). Association between hepatitis C infection and other infectious diseases: a case for targeted screening?. Am J Gastroenterol.

[13] Baddley JW, Perfect JR, Oster RA, Larsen RA, Pankey GA, Henderson H, et al ( 2008). Pulmonary cryptococcosis in patients without HIV infection: factors associated with disseminated disease. Eur J Clin Microbiol Infect Dis.

[14] Spec A, Raval K, Powderly WG ( 2015). End-stage liver disease is a strong predictor of early mortality in cryptococcosis. Open Forum Infect Dis.

[15] Lin YY, Shiau S, Fang CT ( 2015). Risk factors for invasive Cryptococcus neoformans diseases: a case-control study. PLoS One.

[16] Rohilla R, Meena S, Kaistha N, Krishna Raj A, Gupta P ( 2019). Disseminated cryptococcosis and hepatitis C virus infection: A fatal co-infection. Curr Med Mycol.

[17] Shorman M, Evans D, Gibson C, Perfect J ( 2016). Cases of disseminated cryptococcosis in intravenous drug abusers without HIV infection: A new risk factor?. Med Mycol Case Rep.

[18] Spies FS, de Oliveira MB, Krug MS, Severo CB, Severo LC, Vainstein MH ( 2015). Cryptococcosis in patients living with hepatitis C and B viruses. Mycopathologia.

[19] Chang CC, Harrison TS, Bicanic TA, Chayakulkeeree M, Sorrell TC, Warris A, et al ( 2024). Global guideline for the diagnosis and management of cryptococcosis: an initiative of the ECMM and ISHAM in cooperation with the ASM. Lancet Infect Dis.

[20] Ngan NTT, Flower B, Day JN ( 2022). Treatment of cryptococcal meningitis: How have we got here and where are we going?. Drugs.

